# Consistent Individual Differences Drive Collective Movements in a Tibetan Macaque Group

**DOI:** 10.3390/ani14101476

**Published:** 2024-05-15

**Authors:** Sen Ren, Shenqi Liu, Wenkai Sun, Lei Gao, Lei Ren, Jiahui Liu, Weiqi Zhang, Dongpo Xia, Binghua Sun, Jinhua Li, Xi Wang

**Affiliations:** 1 School of Resources and Environmental Engineering, Anhui University, Hefei 230601, China; 2 International Collaborative Research Center for Huangshan Biodiversity and Tibetan Macaque Behavioral Ecology, Hefei 230601, China; 3 School of Life Sciences, Anhui University, Hefei 230601, China; 4 School of Life Sciences, Hefei Normal University, Hefei 230601, China

**Keywords:** personality, leader, follower, collective movement, Tibetan macaque

## Abstract

**Simple Summary:**

Do nonhuman primates, such as Tibetan macaques, exhibit personalities that influence their collective movements? In this study, we not only confirmed the presence of three personality types in Tibetan macaques but also found that individuals with higher sociability scores, higher rank, or lower anxiousness scores were more likely to initiate successful collective movement. We found that macaques with lower anxiousness scores or higher rank attracted more followers, and that higher-rank individuals tended to join movements earlier. Moreover, individuals with higher sociability and boldness scores exhibited shorter joining latency in group movements. These findings provide valuable insights into how personality influences collective movement in nonhuman primates.

**Abstract:**

Collective movement has emerged as a key area of interest in animal behavior. While individual differences are often viewed as a potential threat to group cohesion, growing evidence suggests that these differences can actually influence an animal’s behavior as an initiator or follower during collective movements, thereby driving the group‘s movement and decision-making processes. To resolve the divergence, we asked how personality can affect the dynamics of collective movements in one group of free-ranging Tibetan macaques (*Macaca thibetana*) in Huangshan, China. We assessed individual personality using principal component analysis and applied the generalized linear mixed model and linear mixed model to examine the influence of personality on decision making during collective movements. Our findings reveled three distinct personality types among Tibetan macaques: sociability, boldness, and anxiousness. Individuals with higher sociability scores and rank, or those with lower anxiousness scores, were more likely to initiate successful collective movements. Older individuals were less successful in initiating movements compared to young adults. Leaders with lower anxiousness scores or higher rank attracted more followers, with females attracting larger groups than males. As for followers, individuals with higher rank tended to join the collective movement earlier. Additionally, individuals with higher sociability or boldness scores had shorter joining latency in collective movement. Finally, there was a longer joining latency for middle-aged adults compared to young adults. These results suggest that individual differences are a potential driver of collective movements. We provide some insights into the relationships between personality and decision making in Tibetan macaques.

## 1. Introduction

For many social animals, group living is a fundamental aspect of their life history. However, differences in age, sex, reproductive status, and dominance rank among group members can result in varying motivations and needs for survival [1]. Consequently, conflicts of interest may arise within the group, which can weaken cohesion and lead to a group split-up, so group members may lose some of the advantages of group living [2,3]. To maintain group cohesion, social animals need to engage in decision-making processes that facilitate behavioral alignment and consensus among members [4,5].

In the context of foraging, group members collectively or individually need to decide, for example, the timing of departure from a location and the new travel direction of destination, for which finding a consensus among group members can be complicated [6,7]. The mechanisms by which these decisions are formulated and consensus is reached are significantly influenced by the behavioral characteristics of the individuals comprising the group. This leads us to consider the concept of personality in animal behavior, which is characterized by the consistent individual differences in behavioral that persist over time and across various contexts [8,9]. In a recent study, it was found that group member personalities can influence not only consensus and cooperative decision making, but that they also play a key role in promoting collective movement through more efficient task allocation [10].

For instance, the success of honeybee colonies is significantly bolstered by the behavioral diversity among their members. Colonies with a mix of more diverse foraging behaviors outperform those with less variety, illustrating the advantages of personality differences in group foraging efficiency [11]. Similarly, in various taxa, groups composed of a mix of shy and bold individuals demonstrate superior performance in division of labor compared to homogenous groups. This diversity in personalities contributes to a complementary skillset that can enhance group members’ ability to respond to environmental or social changes [12].

Recent theoretical work has proposed that leadership tendencies may relate to variation in individual-level personality traits [13]. If so, then such consistent differences in personality may help characterize individuals’ roles in collective decision making. For example, exploratory homing pigeons (*Columba livia*) show a high propensity to lead a group movement and reach safe destinations more rapidly than less exploratory individuals [14]. Similarly, in captive barnacle geese (*Branta leucopsis*), bold individuals tended to make asocial decisions and were less likely to use social information [15], though this effect was dependent on group size [16]. Therefore, the personality of boldness seems to make an individual more likely to assume a leadership role within a group.

Shy individuals within a group may not take on leadership roles as frequently as bold individuals, instead choosing to follow the decisions of bolder individuals or the collective group consensus [17]. For example, in pairs of tank-housed three-spined sticklebacks (*Gasterosteus aculeatus*), shy individuals tended to follow their partner’s decisions, although they also had the capacity to enhance leadership traits in bolder individuals [18]. Thus, while shy individuals may not take on leadership roles as frequently as other personality types, they still contribute to the decision-making process by engaging in consensus-building activities [19]. Their involvement is essential for maintaining the group‘s consensus and cohesion.

Most previous studies have typically focused on a narrow range of personality dimensions in species, mainly examining the bold–shy continuum, potentially overlooking the complexity of the mechanisms underlying decision making in species. The majority of the research exploring the impact of personality on decision making has been conducted within captive or experimental environments. Therefore, our understanding of how personality affects intragroup social interactions in the wild, where environments are dynamic and group compositions are naturally occurring, is limited. The intricate behaviors of wild animals, shaped by dynamic ecological and social factors, present significant challenges for our understanding of how personality influences decision making in natural contexts. Therefore, there is an urgent need to explore how consistent individual differences impact collective movement among primate groups in natural environments. In this study, we explored how individual personality consistently impacted collective movement decisions in wild Tibetan macaques (*Macaca thibetana*).

The Tibetan macaque is a highly social species that lives in multimale–multifemale groups, which typically consist of several adult males, adult females, and their offspring. These groups are characterized by a well-defined hierarchy, which is primarily shaped by aggressive and submissive behaviors exhibited among individuals. Additionally, this species exhibits significant behavioral differences between individuals [20]. Previous research has shown that Tibetan macaques have different types of personality, such as sociability and boldness [21]. To examine the personalities within a group of Tibetan macaques and to determine the specific personality associated with leadership, as well as how individuals with different personalities contribute to the group in terms of joining latency and order of joining movements, we tested three predictions: (1) individuals scoring higher in sociability are more likely to initiate successful movement and attract more followers; (2) individuals with higher anxiousness scores would join the movement later; (3) individuals with higher boldness scores would join a collective movement faster than individuals with lower boldness scores.

## 2. Materials and Methods

### 2.1. Study Site and Subjects

We conducted this study from July 2022 to June 2023 in the Valley of the Wild Monkeys, Mt. Huangshan National Reserve in Anhui, China (30°29′ N, 118°10′ E). This location is a UNESCO World Heritage site, classified as such for biodiversity and cultural reasons, and is a popular tourist destination.

The study group of Tibetan macaques was known as Yulinkeng 1 (YA1), which had been continuously observed since 1986. Suomi showed that primate personalities stabilize by adulthood [22]. Therefore, 27 adult monkeys in YA1 were selected for the study (Table 1). All 27 adults could be identified by their unique physical characteristics such as face shape, body appearance, or hair color [20].

The young adult class is defined by individuals at the beginning of their mature phase, with females aged five years and males at seven years. This stage is distinguished by having brown fur and frequent daily activity. Once female individuals give birth, they are quickly accepted by the group. In contrast, male Tibetan macaques may reach sexual maturity at five years old with normal ejaculatory capabilities, but their social status is earned through personal efforts, which is different from that of females. Male macaques must wait until they have grown to a certain size and have the physical prowess to compete for and secure a position within the group before they are fully recognized as adults [20]. In many primate species, male adulthood is typically delayed by two to three years compared to females due to these social and physical maturation differences [23]. Middle-aged individuals, aged between ten and fifteen years, exhibit a darker brown fur and are larger in size compared to the young adults. They often display more thick facial hair and a stable social status within the group. The old adult class includes individuals older than fifteen years, with fur that may appear dark brown or black, and frequently experiences hair loss. Old individuals spend more time resting, move more slowly, and exhibit less activity overall, reflecting the typical age-related decline in vitality [20]. Our team maintains detailed birth records for the study group. For immigrate individuals, we estimate age class through visual assessment of fur color, body size, and overall physical condition.

During our research, we provided the Tibetan macaques with 3–4 kg of artificially supplied corn at 09:00, 11:00, 14:00, and 17:00 and distributed it widely in a highly visible location to prevent monopolization by a single or small number of high-ranking individuals. As the macaques consumed the corn, they would move from the providing area to the surrounding forest. To accurately record the positions and movement distances of each animal, we set up markers at a distance of 10 m in the potential directions from the bait site to the forest and provisioning area.

### 2.2. Data Collection and Behavioral Definition

The data were collected by one researcher (S.R.) who conducted behavioral observations between approximately 08:00–09:00 and 17:00–18:00 each day. An audio recording device (model News my V03) was utilized for focal animal observation and continuous audio documentation of the daily behaviors of both 12 adult males and 15 females [24]. Each focal observation period was scheduled for a duration of 15 min. If the targeted animal was not observable or disappeared during the recording session, a different individual was chosen at random to continue the observation. In our study, focal observations of the 27 adult monkeys totaled 7290 min (per individual: 270 min). 

For each 15 min interval, we chose to record behaviors that we thought would be tied to personality in some way and that were comparable to previous coding studies of personality in nonhuman primates [25,26,27,28]: (1) Two instances of aggressive and submissive behaviors were noted, encompassing the frequency of aggressive behaviors such as staring, hitting on the ground, flapping, grasping, biting, and chasing, as well as the frequency of submissive behaviors including presents, avoidance, and fleeing. (2) Two instances of behavior resembling anxiety were documented, including the duration of time spent on self-grooming and the frequency of self-scratching bouts. (3) Six behaviors related to group affiliation were recorded: the duration of grooming one or more group members; the duration of being groomed by one or more group members; the frequency of approach behaviors initiated by the focal animal was observed and quantified as the number of instances in which it approached from a distance greater than 3 m to less than 1 m, remaining within this range for more than 3 s; the duration of time spent sitting or lying within 3 m of other group members; the frequency of engaging in a bridge with others; and the duration of solitary sitting serving as an indicator of disaffiliation, occurring when the focal animal was at a distance of over 5 m from any group member. Detailed behavioral definitions are shown in Table 2.

**Figure 1:**
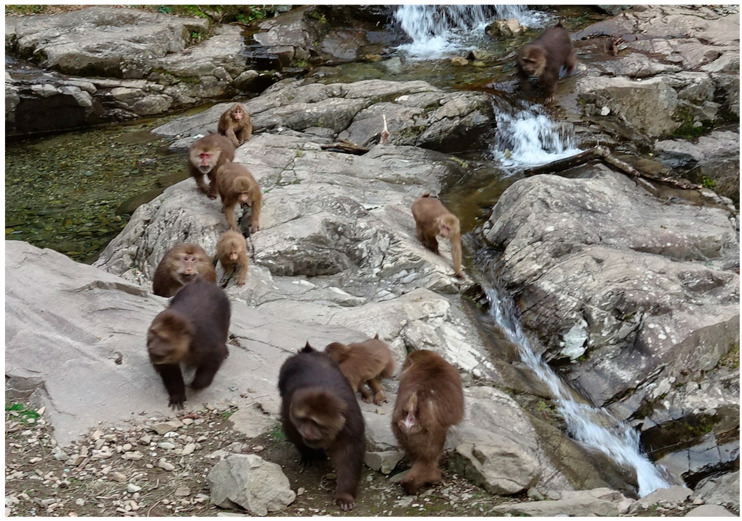
Collective movement in Tibetan macaques.

The processes of collective movements were recorded through an all-occurrences sampling method using video cameras (Canon EOS 550D, Canon, Tokyo, Japan) at set observation sites. This capture method focused on the moments when individuals depart from the provisioning area to return to the forest (Figure 1). Additionally, we set up length points at the provisioning area and along the path from these locations to the forest‘s periphery in order to accurately record the positions and distances covered by each animal. We scored initiation attempts only when there were >18 (two-thirds of adult monkeys) individuals present in the provisioning area. 

The behaviors we recorded during collective movements were initiator and follower of the collective movement, as well as the time of initiation, order of joining, and joining latency of the followers. Table 3 provides a detailed definition of these behaviors [29]. The chaotic movements triggered by conflict events or sexual chases were not recorded [30].

### 2.3. Dominance Hierarchy

We calculated each individual‘s dominance status during the study period by using ad libitum sampling to collect data on the times and direction of aggressive and submissive behaviors among adult individuals. We recorded a total of 803 aggressive and submissive interactions, constructing an aggressive/submissive matrix based on the direction of agonistic interactions given and received. The 28 × 28 aggressive matrix listed aggressors along the vertical axis and recipients along the horizontal, with cell values indicating attack counts. Blanks are used for self-interactions and the diagonal. The submissive matrix, also 28 × 28, switches the roles, with recipients on the vertical and aggressors on the horizontal, maintaining the same blank conventions. Individual dominance rank was then determined using David‘s score (DS) based on the matrix. David’s score was calculated using the following formula [31]:

DS = ∑*P_ij_* + ∑*W_j_* × *P_ij_* − ∑*P_ji_* − ∑*I_j_* × *P_ji_*

Here, *P_ij_* represents the ratio of the number of times that individual *i* defeated individual *j* to the total number of times that individual *i* and individual *j* attacked and submitted; *P_ij_* = *a_ij_*/*n_ij_*, *a_ij_* represents the number of times individual *i* defeated individual *j*; nij represents the total number of aggression–submission bouts between individual *I* and individual *j*. ∑ *W_j_* × *P_ij_* represents the *P_ij_* weighted sum of the individual *i*, *W_j_* represents the sum of all *P_ij_* of individual *j*. ∑*P_ji_* represents the sum of all *P_ji_* of individual *i*. ∑ *I_j_* × *P_ji_* represents the *P_ji_* weighted sum of the individual *i*, *I_j_* represents the sum of all *P_ji_* of individual *j* [32].

The greater the DS, the higher the individual‘s social rank. Aggressive interactions were defined as an individual threatening, chasing, slapping, grabbing, or biting another individual [33]. Submissive behaviors were scored when an individual exhibited a fearful response, such as a fearful grin, cowering, mock leaving, avoidance, fleeing, or screaming.

### 2.4. Standardization of Initiations Movement Counts

We standardized the number of initiations by each individual based on their frequency of identification during the provisioned feeding sessions with the monkeys. This standardization was achieved using the following equation [34]:

*X*′*_i_* = *X_i_*/*T_i_* × 1000

Here, *X*′*_i_* represents the standardized number of initiations for individual *i*. *X_i_* represents the number of collective movement initiations by individual *i*, and *T_i_* denotes the number of times individual *i* was identified by the observer in the feeding area.

### 2.5. Scoring Joining Order and Latency

We scored the joining order of each follower by the assigning position 0 to the first individual to initiate movement. Subsequent individuals were designated position j upon joining, with j reflecting the number of individuals already participating at the time of their join. Consequently, the maximum joining order, *j*_max_, corresponded to *N* − 1, where *N* denotes the total count of adult group members. The joining latency for each follower, denoted as joiner *j*, was determined by the duration between the departure of the preceding joiner (*j* − 1) and the join of joiner j [35].

### 2.6. Personality Analysis

We conducted a principal component analysis (PCA) on a dataset of 27 individuals, each with 10 behavioral measures, using the principal function in R. The data were standardized prior to analysis. We computed eigenvalues for each principal component through 10,000 iterations using parallel analysis and visualized the results with a scree plot. To determine the relevance of each component, we retained components with eigenvalues ≥ 1, indicating their importance in explaining variance. We then employed the prcomp function from the stat package to ascertain the variance accounted for by the retained principal components. To enhancing interpretability, we applied varimax rotation using the principal function to obtain standardized factor loadings and scores with the package psych [36]. Cross-loaded factors were assigned to the component with the highest loading. Personality types were characterized based on the significant loadings of the retained components. 

### 2.7. Statistical Analysis

Correlations between DS, different personality scores, and the amount of initiation data were analyzed using the Pearson correlation coefficient (r) if the data were normally distributed or the Spearman rank coefficient (rs) if the data could not be normalized. To test for differences in sex, we used a t test or a Mann–Whitney U test.

We employed the lme4 package in R to construct generalized linear mixed models (GLMMs) and a linear mixed model (LMM) to examine the influence of individual sex, age, DS, and personality scores on collective movement. For the analysis of collective movement success (a binary outcome), we employed a GLMM with a binomial distribution and a logit-link function. To analyze the number of followers and joining order, we used a GLMM with a Poisson distribution. The joining latency, which was normalized through a square root transformation to meet the assumptions of normality, was analyzed using an LMM. In these models, the initiator‘s identity was specified as a random effect for collective movement success and the number of followers, while the follower‘s identity was considered a random effect for joining order and joining latency. The fixed effects included individual sex, age, DS, and personality scores. Model selection was performed using the MuMIn package in R, based on the corrected Akaike information criterion (AICc) to identify the most suitable models. Models with ΔAICc less than 2 were considered as plausible. The most explanatory model was finally determined according to AICc values and Akaike weights, and we considered the top-level model to be the best model [37]. 

Statistical significance was assessed at a level of α = 0.05.

## 3. Results

### 3.1. Personality Types of Tibetan Macaques

**Figure 2:**
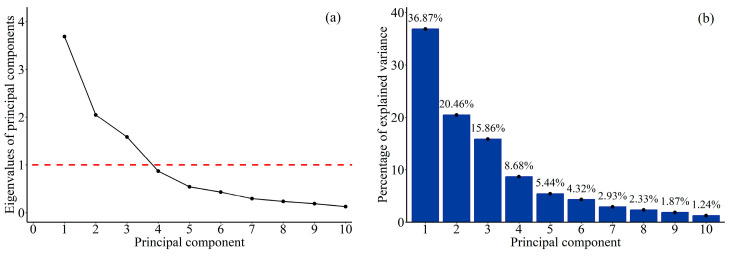
Scree plot of eigenvalues from principal component analysis (**a**) and explained variance by principal components (**b**).

Principal component analysis (PCA) on the ten behaviors recorded from all adult monkeys indicated that the first three principal components (PCs) reached the criteria for a scree plot test, had an eigenvalue above 1, and cumulatively explained 73% of the total variance in the correlation matrix (Figure 2). According to the specific behaviors in Table 4 that loaded onto each PC, individuals that scored higher on component 1 (PC1) spent less time sitting alone and were more likely to approach, groom, and bridge groupmates, as well as spending more time in close proximity to their groupmates. Those with high scores on component 2 (PC2) displayed heightened aggression levels, decreased submission levels, and were more likely to be groomed. Individuals with high scores on component 3 (PC3) exhibited a greater tendency towards self-scratching and self-grooming. From the component factor loadings, we conceptualized PC1 as an individual‘s social approachability, PC2 as confidence and impulsivity, and PC3 as the expression of anxiety. Based on these results, we assigned PC1 as “sociability”, PC2 as “boldness”, and PC3 as “anxiousness”. 

The scores of all 27 adult individuals on four principal components are presented in the Appendix A.

### 3.2. Effects of Sex, David’s Score, and Personality on the Number of Initiations

**Figure 3:**
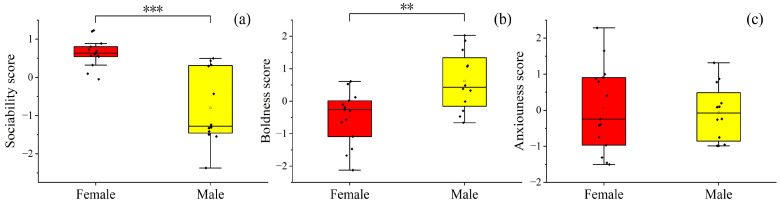
Sex differences in sociability (**a**), boldness (**b**), and anxiousness scores (**c**) among Tibetan macaques. Solid rhombuses represent an outlier in the data, empty square represents the average value of the data. Significant differences: ***: *p* < 0.001; **: *p* < 0.01.

Males had higher David‘s scores than females (*t* = 2.80, *p* < 0.05), as well as higher boldness scores (*t* = 3.39, *p* < 0.01). In contrast, females outscored males in terms of sociability (*Z* = −3.86, *p* < 0.001). No significant sex differences were found in anxiousness scores (*t* = −0.32, *p* > 0.01) or the number of initiations (*t* = −0.41, *p* > 0.05) (Figure 3).

David‘s score was not significantly correlated with sociability (Spearman rank correlation: rs = 0.029, *p* = 0.885) or anxiousness scores (Pearson rank correlation: r = −0.073, *p* = 0.719); however, it was significantly correlated with boldness scores (Pearson rank correlation: r = 0.762, *p* < 0.001). The number of initiations was not significantly correlated with the three personality scores (Spearman rank correlation: rs = 0.124, *p* = 0.537; Pearson rank correlation: r = 0.119, *p* = 0.555; Pearson rank correlation: r = −0.181, *p* = 0.366) or with David’s score (Pearson rank correlation: r = −0.139, *p* = 0.490). Furthermore, there were no significant correlations among the three personality scores (Spearman rank correlation: rs = 0.021, *p* = 0.918; Spearman rank correlation: rs = 0.013, *p* = 0.949; Pearson rank correlation: r = 0.001, *p* = 0.996) (Table 5).

### 3.3. The Influence of Individual Characteristics on Group Movements

In our analysis, we recorded a total of 248 attempts for initiating group movements, with 173 successful attempts and 75 resulting in failure. Through model selection, we identified the following as the most predictive models for different outcomes: for the collective movement success (Age, DS, PC1, PC3, wi = 0.17); number of followers (Age, DS, Sex, PC3, wi = 0.13), joining order (Age, DS, wi = 0.09), and joining latency (Age, PC1, PC2, PC3, wi = 0.11) (Table 6).

Based on the model selection and inference results presented in Table 6, several factors were identified as significant predictors of various outcomes. Regarding collective movement success, individuals with higher sociability scores (relative variable importance, RVI) (RVI = 0.69, β ± SE = 0.50 ± 0.17, *p* < 0.01), lower anxiousness scores (RVI = 1, β ± SE = −0.44 ± 0.17, *p* < 0.01), and higher DS (RVI = 1, β ± SE = 0.00 ± 0.00, *p* < 0.05) were more likely to initiate successful movement. In contrast, old individuals (Age3; RVI = 1, β ± SE = −1.65 ± 0.63, *p* < 0.01) were less likely to initiate successful collective movements compared to young adults. Regarding the number of followers, individuals with higher anxiousness scores (RVI = 0.89, β ± SE = −0.10 ± 0.05, *p* < 0.05) had fewer followers, males (RVI = 0.67, β ± SE = −0.40 ± 0.11, *p* < 0.001) had fewer followers than females, and individuals with higher DS (RVI = 1, β ± SE = 0.00 ± 0.00, *p* < 0.001) had more followers.

In terms of joining order, individuals with higher DS (RVI = 0.87, β ± SE = 0.00 ± 0.00, *p* < 0.05) joined the movement earlier. For joining latency, individuals with higher sociability scores (RVI = 0.54, β ± SE = −0.39 ± 0.18, *p* < 0.01), higher boldness scores (RVI = 1, β ± SE = −0.43 ± 0.14, *p* < 0.01), and middle-aged (Age2; RVI =1, β ± SE = −0.39 ± 0.18, *p* < 0.01) individuals had longer joining latency than young individuals (Table 7).

## 4. Discussion

Based on our collected data and the limitations of our study, we identified at least three personality types within our study group of Tibetan macaques: sociability, boldness, and anxiousness. Behavioral differences between individuals, in conjunction with sex, age, and rank, together influenced their collective movement and decision-making processes of our group.

Our results partially supported the first prediction: individuals with higher sociability scores were more likely to initiate successful collective movements, although they did not attract more followers. The second prediction was not supported as there was no evidence that personality affected the order in which individuals joined collective movements. However, it was noteworthy that individuals with higher rank tended to join in movements earlier. The third prediction was supported by individuals with higher boldness scores exhibiting shorter latency to join movement, a pattern that was also observed in individuals with higher sociability scores.

### 4.1. Sex Differences and Dominance Hierarchy in Personality

Within our study group of Tibetan macaques, we found that sex played a significant role in the expression of these personality traits. Specifically, female Tibetan macaques in our group exhibited higher scores in sociability, while male Tibetan macaques scored higher in boldness, which was consistent with sex differences observed in Asian elephants (*Elephas maximus*) [38]. However, previous studies in primates have reported contrasting findings, with male bonobos (*Pan paniscus*) having higher introversion scores, while females had significantly higher irritability scores [27]. The sex differences in sociability among Tibetan macaques could be accounted for by the different life histories of the sexes. In many primate species, including the Tibetan macaque, males reach adulthood and disperse from their natal groups, resulting in family units that primarily consist of related females [39]. This matriarchal society increases the likelihood of females developing stronger and more intimate associations. The enhanced sociability among females likely reinforces kinship bonds, which are crucial for maintaining group stability and facilitating the care of offspring [40]. In the previous research on personality in the macaques, sex difference was influenced by social style. Specifically, in species with more relaxed social style, females were more friendly than males. Furthermore, in highly despotic species, females exhibited less variation in scores on the dominance dimension, which can be attributed to social style [41].

In male Tibetan macaques, boldness scores were positively correlated with their dominance hierarchy ranks, indicating that bolder individuals were more likely to attain higher ranks. Furthermore, boldness is predictive of increased aggressive behaviors, which are essential for engaging in more aggressive interactions to establish and assert their dominance status within the group. This has been confirmed in several species, such as chimpanzees (*Pan troglodyte*) [42], zebrafish (*Danio rerio*) [43], and rhesus macaques (*Macaca mulatta*) [28]. Consequently, this behavioral strategy was pivotal for securing and maintaining high rank, which in turn provides advantages in accessing resources like food and enhancing mating opportunities. During the mating season, our observations revealed that high-ranking individuals almost monopolized the majority of mating opportunities within the group, with mating behaviors among low-ranking individuals being scarce. This phenomenon has also been observed in other species, including yellow baboons (*Papio cynocephalus*) [44], olive baboons (*Papio ursinus*) [45], and mandrills (*Mandrillus sphinx*) [46], where high-ranking individuals similarly dominate mating resources.

### 4.2. Individual Characteristics and the Initiator of Group Movements

During the observation, we found that leadership was distributed among group members, with all adult individuals successfully initiating movement at least once (Table 1). Noticeably, there was variation among individuals in the frequency of movement initiation, but this did not correlate with their personalities. This result was not consistent with studies on domestic horses [19] and three-spined sticklebacks [18], which found that bolder individuals initiated more movements. The behavior attributed to their lower responsiveness to others and a greater inclination towards exploration, which made it easier to make decision to move away from the group [47]. In some species, the initiation of movements has often been correlated with individual motivations, such as lactating females that are driven by increased energy requirements and the imperative to protect their infants, taking the lead in initiating movements. This pattern has been documented in species such as white-handed gibbons (*Hylobates lar*) [48] and ring-tailed lemurs (*Lemur catta*) [49], where lactating females have been observed to take a proactive role in prompting collective movements.

In our study group, individuals with higher sociability scores exhibited a greater tendency to successfully initiate collective movements. These scores reflect increased rates of social engagement within the group. This was similar to previous research that suggests individuals with higher eigenvector centrality, an indicator of social prominence based on affiliative behaviors such as grooming and proximity, were better at leading group movements [50] and emitted more frequent visual communication during collective decision making [29]. The relationship between close social connections and the success in initiating movements reinforced the concept of “distributed leadership” among Tibetan macaques, which may be correlated with the high social tolerance within Tibetan macaque groups. Species within the macaque genus that exhibit high social tolerance have been found to possess superior cognitive skills, which facilitate improved cooperation and communication [51]. Sueur and Petit‘s comparison of leadership in Tonkean macaques (*Macaca tonkeana*) and Rhesus macaques highlights the influence of social tolerance on group movement. In Tonkean macaques, with higher social tolerance, group movements were a collaborative effort. By contrast, in Rhesus macaques with lower social tolerance, group movements were often led by high-ranking males [35]. Highly anxious individuals, characterized by elevated anxiety levels and tense emotions, frequently lacked confidence [52]. This lack of confidence prevents their ability to make collective decisions and gain the following of other group members. Consequently, their initiations for collective movements were less likely to succeed. This is also a key reason for the limited followership in their movements, as their past unsuccessful attempts may have led to a reduced probability of group members responding to their leadership.

The decreased probability of successful collective movement initiation by old individuals relative to their young adult counterparts may have been due to a decline in their capacity to process decision-making information [53]. Furthermore, the concept of homophily provided a plausible explanation for the challenges faced by old individuals in initiating successful collective movements. Homophily posits that individuals are more likely to form associations and bonds with others that possess similar characteristics [54]. In the context of age, our study group, characterized by a mere two individuals of old age, naturally limited the potential for age-related homophily. The lack of age-similar individuals within the group likely contributed to weaker group cohesion and less sample size of potential followers when old individuals attempt to initiate collective movements. Consequently, the lower probability of successful movement initiation by old individuals may, in part, have been explained by the reduced opportunities for age-related homophily.

In previous research, it was observed that females were more adept at using vocal communication to recruit a greater number of followers than males when initiating group movements. This proficiency in vocal signaling appeared to be a key factor in the higher follower recruitment by females, highlighting their important role in leading collective actions within the group [55]. A similar phenomenon was noted in Barbary macaques, further supporting this pattern of behavior [56]. Additionally, as Tibetan macaques are seasonal breeders, the period of peak sexual activity in females was characterized by the formation of temporary mating relationships with males. During this time, males maintain close proximity to females, frequently adjusting their positions to trail behind them. This behavior has also been observed in other species, such as crab-eating macaques (*Macaca fascicularis*) [57] and lion-tailed macaques (*Macaca silenus*) [58]. This behavior served a dual purpose: it deterred other males from approaching the sexually active females, thereby increasing the likelihood of successful mating for the attending male, and it ensured a high level of mate guarding. Mate guarding was a critical strategy for males to secure reproductive success by reducing the chances of sperm competition and increasing the probability of the transmission of their genes [59]. In our study group, we observed a significant effect of an individual‘s rank on the number of followers after initiating movements. Specifically, higher-ranking individuals were able to attract a greater number of followers. This suggests that while rank did not affect the initiation of movements, it did play a critical role in the subsequent recruitment of followers. We proposed that the greater number of followers recruited by dominant individuals may have been attributed to the group members’ inclination to form social alliances with them. These alliances could serve to mitigate aggression from higher-ranking individuals [60] or to collectively defend against external threats.

### 4.3. Individual Characteristics and Joining Process

In the context of collective movement, certain advantages could be gained from the front position of an individual within the progression order. For example, dominant males or lactating females were at the front of the progression, where they had priority access to food resources and could decide which direction to take (e.g., yellow baboons, [61]. These individuals also exhibited higher mate appeal and tended to integrate into the movement at an earlier stage during collective decision making, thereby potentially expediting and facilitating the onset of group movements. However, in the study of chimpanzees, road-crossing, a challenge introduced by human activity, presents a new scenario that requires chimpanzees to respond flexibly to varying perceived risks. During such hazardous journeys, certain positions may offer more benefits than others, often depending on factors such as age and sex. Adult males, who are typically less apprehensive and more physically formidable than other group members, assume forward and rearward positions, while adult females and juveniles take up the more sheltered middle positions [62]. These strategies reflect the species’ adaptation to new environmental challenges and provide insights into the social organization of chimpanzees during potentially dangerous situations. The differences observed in Tibetan macaques may be attributed to their habitat, which has fewer natural predators, leading to less need for protective strategies during movement.

The latency in joining collective movement was significantly linked to an individual‘s sociability and boldness scores. Individuals with high sociability scores frequently engaged in social interactions, which often placed them in close proximity to other group members. As a result, they were more likely to promptly notice when a movement was initiated by nearby individuals, leading to a shorter joining latency. In addition, those with high boldness scores showed greater efficacy in collective information processing and decision making, as well as increased sensitivity to environmental changes [63]. This heightened responsiveness contributed to a reduced latency in their joining time when responding to the initiator‘s movement. The longer joining latency of middle-aged individuals compared to young adults may have been attributed to the fact that the studied group was of a free-range type, where food and resources were likely to be relatively abundant. Middle-aged individuals may not have a strong incentive to take unnecessary risks.

## 5. Conclusions

In conclusion, our study within a group of Tibetan macaques has highlighted the roles of personality, sex, age, and social rank in driving collective movement behaviors. These findings provide valuable insights into the underlying mechanisms of group dynamics and the importance of individual differences in coordinating group activities. However, it is important to acknowledge the limitations of our study, including the relatively short sampling duration of 4.5 h per individual. Personality traits, which are based on a series of behaviors that can change or be adapted to specific situations and can be highly variable depending on the moment, may not have been fully captured by the short sampling duration. Future research would aim to increase the duration of individual observations to obtain a more stable assessment of personality traits.

Furthermore, through daily observations, we have noted that there may be inherent grammatical rules in the sequence of individuals joining collective movements. For example, individual A consistently follows individual B, rather than vice versa, or a fixed sequence of individuals CDEF consistently participate in the movement. In our future research, we plan to conduct a detailed examination of these temporal sequences to identify any recurring patterns. Additionally, we aim to investigate the factors that may influence these grammatical rules, such as behavioral differences among individuals, kinship, and the strength of social connections. 

## Figures and Tables

**Table 1:** Characteristics of focal animals from the YA1 group in the observation.

Individual	Sex	Date of Birth/Immigrate	Age Class	David’s Score	Number of Initiation Attempts	Standardized Number of Initiations
YL	Male	24 August 2021 (I)	1	203.32	10	41.84
YXK	Male	14 February 2013	2	178.79	14	60.87
WM	Male	20 November 2018 (I)	2	123.33	12	67.42
TQ	Male	27 November 2018 (I)	2	123.26	13	54.39
YXX *	Female	8 May 2010	2	101.62	22	99.10
YH	Female	2003	3	77.87	14	62.78
NM	Male	8 August 2021 (I)	2	77.81	12	59.70
TQS	Male	4 May 2015	1	28.81	6	36.36
YCY *	Female	12 March 2009	2	17.62	21	98.59
YXY *	Female	19 April 2015	1	14.32	12	64.17
BHZ	Male	15 October 2021 (I)	2	2.37	9	72.58
YCH *	Female	October 2015 (I)	2	−13.48	5	28.41
WS	Male	4 January 2022 (I)	2	−20.14	2	58.82
LB	Male	22 November 2021 (I)	2	−22.16	2	41.67
TXH	Female	2009	2	−23.18	12	65.93
DZ	Male	3 November 2021 (I)	1	−23.45	5	49.02
DB	Male	September 2021 (I)	1	−24.28	3	34.48
YCL	Female	15 September 2012	1	−27.46	5	30.86
TQG	Female	18 June 2017	1	−36.96	8	50.63
QT	Male	5 September 2021 (I)	1	−74.14	4	74.07
TQL *	Female	25 March 2013	1	−77.01	14	74.87
TFH	Female	27 March 2016	1	−80.78	2	26.32
TH *	Female	2003	3	−84.01	5	28.57
TXX	Female	17 March 2008	2	−99.00	9	37.66
THY	Female	8 April 2009	2	−107.97	17	96.59
THX	Female	19 April 2012	2	−116.79	6	78.95
TQY	Female	4 March 2016	1	−118.30	4	23.95

The higher the David’s scores, the higher the rank. (I): the immigration date of individuals. * Present females with offspring less than 6 months old.

**Table 2:** Behavioral definitions for personality assessment in Tibetan macaques [20].

Catalog	Behavior	Definition
Aggression	Stare	An individual looks directly at another individual with its eyes wide open and with its shoulders raised for about 3–5 s.
	Hitting on the ground	An individual supports with one hand on the ground; the other flaps the ground and stares to the other individual.
	Flap	One individual uses its hands or arms to strike or wave at another individual.
	Grasp	One individual grasps the hair of the body, face, or neck of another one, or it may simply grab its ears, shaking it back and forth a few times before letting go.
	Bite	The performer grabs the receiver tightly, preventing him/her from fleeing, and bites the recipient vigorously.
	Chase	One individual runs rapidly after another individual.
Submission	Present	One individual displays his or her rump to another.
	Avoidance	The individual shows a fearful face and turns his/her body to the one attacking or approaching it and poses as if fleeing, showing a fearful posture.
	Flee	The individual rapidly moves in the opposite direction from the attacker.
Anxiety	Self-grooming	Picking through and/or slowly brushing aside fur with one or both hands.
	Self-scratching bouts	Movement of the hand or foot, during which the fingertips are drawn across fur or skin.
Affiliation	Grooming given	One individual uses his/her hand or mouth to manipulate the fur of another individual; sometimes, the groomer may pick out small items in the fur and eat them.
	Grooming received	The fur of an individual is manipulated by another individual’s hand or mouth.
	Approach	One individual close to other individual from a distance greater than 3 m to less than 1 m.
	Proximity	Two or more individuals are sitting or lying within 3 m of one another.
	Bridging	A complex sequence of behavior in which an individual approaches another, alternating glances at the receiver and an infant that is carried by either the approacher or the approached. The pair holds the infant between them and simultaneously licks the infant’s genitals or body while teeth-chattering vigorously.
	Sit alone	One individual sitting alone with no other individuals within 5 m.

**Table 3:** Behavioral definitions during Tibetan macaques’ collective movements (adapted from Tang [29,30]).

Catalog	Definition
Initiator	The first individual to move a distance more than 10 m within a time under 30 s.
Follower	Any individual seen moving away from the group more than 5 m within 45° in the direction as the initiator and within 5 min after the initiation of the first initiated individual.
Successful movement	When the total number of participants, including the initiator, is equal to or more than 3, it is documented as a successful collective movement.
Termination of joining	When the last individual joins and no more individual join the movement within the following five minutes.

**Table 4:** Standardized, varimax-rotated factor loadings of behaviors in principal component analysis (PCA).

Behavior	PC1	PC2	PC3
	Sociability	Boldness	Anxiousness
Sit alone (D)	**−0.92**	−0.08	0.12
Proximity (D)	**0.70**	0.39	−0.28
Approach (F)	**0.86**	0.17	0.11
Bridging (D)	**0.79**	0.09	−0.11
Grooming given (D)	**0.78**	0.28	0.32
Grooming received (D)	0.25	**0.58**	0.05
Aggression (F)	0.26	**0.78**	0.20
Submission (F)	0.28	**−0.80**	−0.09
Self-grooming (D)	0.14	−0.32	**0.85**
Self-scratching bouts (F)	−0.19	0.33	**0.87**

Bold typeface indicates the strongest factor loadings. D: duration; F: frequency.

**Table 5:** Results of correlations between David’s score, personality, and number of initiations.

	Sociability	Boldness	Anxiousness	Number of Initiations
David’s score	rs = 0.029	r = 0.762	r = −0.073	r = 0.139
	*p* = 0.885	***p* < 0.001**	*p* = 0.719	*p* = 0.490
Sociability		rs = 0.021	rs = 0.013	rs = 0.124
		*p* = 0.918	*p* = 0.949	*p* = 0.537
Boldness			r = 0.001	r = 0.119
			*p* = 0.996	*p* = 0.555
Anxiousness				r = −0.181
				*p* = 0.366
Number of initiations				

Bold *p*-values represent statistically significant differences.

**Table 6:** Model selection for models used to explain the effect of age, sex, DS, and personality score in Tibetan macaques on collective movements.

Model	df	AICc	∆AICc	wi
Collective movement success				
Age + DS + PC1 + PC3	7	295.14	0.00	0.17
Age + DS + Sex + PC3	7	295.42	0.28	0.15
Number of followers				
Age + DS + Sex + PC3	7	1009.83	0.00	0.13
Age + DS + PC1 + PC2 + PC3	8	1010.47	0.64	0.10
Joining order				
Age + DS	5	2523.25	0.00	0.09
DS	3	2523.57	0.32	0.08
Joining latency				
Age + PC1 + PC2 + PC3	8	3578.44	0.00	0.11
Age + Sex + PC2 +PC3	8	3578.55	0.12	0.10

Complete models contain Age, DS (David’s score), Sex, PC1 (sociability score), PC2 (boldness score), and PC3 (anxiousness score) as fixed effects; df = the degree of freedom, AICc = Akaike information criterion values, ∆AICc = difference between the AICc value of the specified model and the optimal model, wi = model weight.

**Table 7:** Results from the best GLMM and GLM analyses examining whether age, sex, DS, and personality score significantly predict collective movement in Tibetan macaques.

Variable	Estimate	SE	Z	*p*
Collective movement success				
Intercept	0.95	0.27	3.51	0.000 ***
PC1	0.50	0.17	2.85	0.004 **
PC3	−0.44	0.17	−2.65	0.008 **
Age2	−0.31	0.34	−0.92	0.358
Age3	−1.65	0.63	−2.63	0.008 **
DS	0.00	0.00	2.24	0.025 *
Number of followers				
Intercept	0.99	0.08	11.82	0.000 ***
PC3	−0.10	0.05	−2.10	0.036 *
Age2	0.10	0.09	1.07	0.285
Age3	−0.31	0.18	1.73	0.084
Sex (Male)	−0.40	0.11	−3.60	0.000 ***
DS	0.00	0.00	4.33	0.000 ***
Joining order				
Intercept	0.98	0.04	26.22	0.000 ***
Age2	0.05	0.05	1.05	0.294
Age3	−0.12	0.09	−1.36	0.175
DS	0.00	0.00	−2.46	0.014 *
Joining latency				
Intercept	5.51	0.23	24.42	0.000 ***
Age2	0.99	0.31	3.21	0.001 **
Age3	1.00	0.51	1.96	0.051
PC1	−0.39	0.18	−2.14	0.033 *
PC2	−0.43	0.14	−3.14	0.002 **
PC3	0.22	0.14	1.58	0.114

Age2: middle-aged, male and female ≥ 10~15 years old; Age3: old, male and female ≥ 15 years old. Significant differences: ***: *p* < 0.001; **: *p* < 0.01; *: *p* < 0.05.

## Data Availability

The data that support the findings of this study are available from the corresponding author, upon reasonable request.

## Appendix A. The Principal Component Scores for Each Adult Monkey in This Study

**Individual**	**Sociability**	**Boldness**	**Anxiousness**
YXX	0.80	−0.25	−0.43
YH	1.23	0.53	−1.31
YCY	1.20	−0.57	−0.24
YXY	0.89	0.12	−1.50
YCH	0.72	−0.10	2.28
TXH	0.69	0.61	1.00
TQL	0.61	0.01	0.91
TH	0.80	−0.25	0.79
YCL	0.64	−0.30	1.65
TXX	−0.05	−1.09	−1.46
THY	0.57	−0.19	−0.75
THX	0.10	−0.64	0.88
TQY	0.56	−1.47	−0.97
TFH	0.54	−2.12	0.40
TQG	0.32	−1.67	−0.39
YL	0.43	2.03	−0.99
YXK	0.33	1.86	0.10
DZ	−1.25	0.48	−0.75
NM	0.50	1.08	0.19
TQ	0.30	1.58	0.78
WM	−1.50	0.38	0.09
BHZ	−1.31	−0.01	0.87
TQS	−0.43	1.09	−0.24
LB	−1.55	0.33	−0.95
WS	−1.42	−0.66	−0.26
DB	−2.38	−0.47	1.31
QT	−1.33	−0.30	−0.99
